# Modeling of flaxseed protein, oil content, linoleic acid, and lignan content prediction based on hyperspectral imaging

**DOI:** 10.3389/fpls.2024.1344143

**Published:** 2024-02-12

**Authors:** Dongyu Zhu, Junying Han, Chengzhong Liu, Jianping Zhang, Yanni Qi

**Affiliations:** ^1^ College of Information Science and Technology, Gansu Agricultural University, Lanzhou, China; ^2^ Crop Research Institute, Gansu Academy of Agricultural Sciences, Lanzhou, China

**Keywords:** hyperspectral imaging, flaxseed, protein, oil content, linoleic acid, lignan

## Abstract

Protein, oil content, linoleic acid, and lignan are several key indicators for evaluating the quality of flaxseed. In order to optimize the testing methods for flaxseed’s nutritional quality and enhance the efficiency of screening high-quality flax germplasm resources, we selected 30 flaxseed species widely cultivated in Northwest China as the subjects of our study. Firstly, we gathered hyperspectral information regarding the seeds, along with data on protein, oil content, linoleic acid, and lignan, and utilized the SPXY algorithm to classify the sample set. Subsequently, the spectral data underwent seven distinct preprocessing methods, revealing that the PLSR model exhibited superior performance after being processed with the SG smoothing method. Feature wavelength extraction was carried out using the Successive Projections Algorithm (SPA) and the Competitive Adaptive Reweighted Sampling (CARS). Finally, four quantitative analysis models, namely Partial Least Squares Regression (PLSR), Support Vector Regression (SVR), Multiple Linear Regression (MLR), and Principal Component Regression (PCR), were individually established. Experimental results demonstrated that among all the models for predicting protein content, the SG-CARS-MLR model predicted the best, with and of 0.9563 and 0.9336, with the corresponding Root Mean Square Error Correction (RMSEC) and Root Mean Square Error Prediction (RMSEP) of 0.4892 and 0.5616, respectively. In the optimal prediction models for oil content, linoleic acid and lignan, the ${\text{R}}_{\text{p}}^{2}$ was 0.8565, 0.8028, 0.9343, and the RMSEP was 0.8682, 0.5404, 0.5384, respectively. The study results show that hyperspectral imaging technology has excellent potential for application in the detection of quality characteristics of flaxseed and provides a new option for the future non-destructive testing of the nutritional quality of flaxseed.

## 1 Introduction

Flax (Linum usitatissimum) occupies an important position in oil and fiber crops (Oomah, 2001). According to its application scope, it is divided into fiber, oil, and fiber oil three (Zhang et al., 2011). Flaxseed is rich in essential omega-3 fatty acids, α-linolenic acid, and linoleic acid is recognized as a major source of high-quality proteins, lignan, lipids, and dietary fiber (Katare et al., 2012; Goyal et al., 2014), has a positive effect on the human diet and health, and its processed products in the world have a wide range of demand, belonging to the typical functional crops.

Currently, protein content in flaxseed is primarily determined through chemical analytical methods, like Kjeldahl nitrogen determination (Mueller et al., 2010; Yao et al., 2022). This method first requires drying and grinding of the sample, adding chemical reagents and heating, followed by distillation, titration treatment with a standard hydrochloric acid solution, and finally, a comprehensive calculation of the protein content results based on the values obtained from each process. Other methods for determining oil content often involve organic solvent extraction, while the quantification of linoleic acid and lignan is typically carried out using high-performance liquid chromatography (Meng et al., 2001; Feng et al., 2016). These traditional biochemical determinations of flaxseed nutrient content must be operated by professionals to complete the handling and operation process, which is both complex and professional, not only time-consuming and labor-intensive but also destructive to the sample and incidentally produces chemical pollution. To enhance the efficiency of screening high-quality flax germplasm resources, it is imperative to identify an accurate, rapid, and non-destructive method for assessing protein, oil content, linoleic acid, and lignan content.

HSI technology simultaneously captures the target’s spatial characteristics and spectral information, effectively combining image and spectral data (Xiang et al., 2022). The spectral properties of an object are closely related to its intrinsic physicochemical properties, and the differences in the composition and structure of substances result in the selective absorption and emission of photons of different wavelengths within the substance. Presently, HSI serves as a non-destructive and expeditious analytical tool across various domains, including medical diagnosis (Bjorgan and Randeberg, 2015), food industry (Ma et al., 2019), fruit damage and disease detection (Tian et al., 2020; Yadav et al., 2022; Jiang et al., 2023), and plant seed analysis (Zhu et al., 2019). HSI has proven to be an effective technique for non-destructive seed quality testing by many scholars. For instance, Tu et al. (Tu et al., 2022) used HSI to detect similar maize authenticity. Zou et al. (Zou et al., 2023) employed HSI to gauge peanut seed vigor. In addition, Yoo et al. (Yoosefzadeh-Najafabadi et al., 2021) used HSI for soybean yield prediction. Zhang et al. (Zhang et al., 2022) Used HSI to detect hybrid wheat seed purity. Lu et al. (Lu et al., 2022) ingeniously combined HSI with deep convolutional generative adversarial networks to predict the oil content of individual corn kernels. Yu et al. (Yu et al., 2016) effectively measured fat content in peanuts ( ${\text{R}}_{\text{p}}^{2}$ = 0.84 and SEP = 1.88) and Ma et al. (Ma et al., 2021) further devised a streamlined model for the non-destructive assessment of protein content in rice, achieving notable success ( ${\text{R}}_{\text{p}}^{2}$ = 0.8011 and RMSEP = 0.52). All of these studies demonstrated the feasibility of detecting seed quality based on HSI. However, few studies have been reported on HSI detection of the internal quality of flaxseed. Leomara Floriano Ribeiro et al. employed infrared reflectance spectroscopy and multivariate correction to predict linolenic and linoleic acid content in flaxseed, achieving prediction sets with ${\text{R}}_{\text{p}}^{2}$ values as high as 0.90 and 0.86 (Ribeiro et al., 2013). While this method achieves high accuracy, it is limited to determining the content of linolenic and linoleic acids in only two types of flaxseed. Currently, with over 5,000 flax varieties in commercial cultivation, each exhibiting significant variations in nutrient composition, the method lacks generalizability and stability, rendering it ineffective for the determination of other species. Party Zhao et al. used near infrared analysis technology to determine the quality of flax germplasm resources, and Ye Jiali et al. used non-destructive near infrared spectroscopy to quantitatively analyze the content of flax seed protein, linolenic acid, and lignan (Dang and Zhao, 2008; Ye et al., 2021). The above three non-destructive tests on the nutritional quality of flaxseed are used in the infrared spectrometer wavelength range of 1100-2500 nm, 900-1700 nm, and 1000-2499 nm. The wavelength range of the imaging instrument, although high precision, the cost is expensive; the processing and operation of the process are both complex and professional, and it is not only not applicable to field operations but also general scientific researchers and flax planting researchers cannot be realized. In addition, these methods might not completely capture the internal features of the specimen, and they are solely employed to acquire spectral details from a solitary point source. The uniformity of the sample distribution consistently influences this and may not be the optimal selection. (Ozaki, 2021; Hu et al., 2023).

This project is dedicated to studying the 400-1000nm spectral range of flaxseed nutritional quality detection to fill the existing band range of research gaps. The spectral range of imaging instruments is relatively common and inexpensive. General researchers and flax planting researchers can easily buy and use. This study simultaneously analyzed the flaxseed protein, oil content, linoleic acid, and lignans’ 4 nutrient content. Common reports of up to 3 nutrients have been analyzed in the literature. From the results of the literature available from multiple sources, it is the first time that the content of four nutrients was analyzed simultaneously. Additionally, comprehensively detecting multiple indicators of flaxseed allows for a more integrated assessment of its quality. Various nutrients in flaxseed are interconnected; therefore, solely predicting a single nutritional indicator is insufficient for quality measurement. Practical significance is achieved only through a simultaneous and comprehensive evaluation of several indicators. This integrated research approach contributes to a more thorough, systematic understanding and utilization of the potential value of flaxseed. Thus, this study seeks to establish a non-destructive and expeditious method utilizing HSI for detecting protein content, oil content, linoleic acid, and lignan in flaxseed. The primary research objectives encompass: (1) establish a PLSR prediction model of flaxseed protein content based on raw and preprocessed spectra and determine the optimal preprocessing method through model evaluation; (2) construct prediction models for flaxseed protein, oil content, linoleic acid, and lignan based on distinctive wavelengths extracted by SPA and CARS, using PLSR, PCR, SVR, and MLR. The selection of the optimal prediction model for flaxseed protein, oil content, linoleic acid, and lignan relies on ${\text{R}}_{\text{p}}^{2}$ and RMSEP values to achieve swift, non-destructive, and precise nutritional quality prediction; (3) identifying characteristic spectral bands pertinent to protein, oil content, linoleic acid, and lignan in flaxseed based on the most effective model.

## 2 Materials and methods

### 2.1 Experimental materials

As shown in **Table 1**, thirty flaxseed varieties, extensively cultivated in Northwest China, were selected for the study. Seed samples were obtained from the Gansu Academy of Agriculture’s Crop Institute. All the varieties were harvested in 2022 from the experimental field of Lanzhou New District, Gansu Province, China, situated at an altitude of 1520 m above sea level (103°72’E,36°03’N). To limit water absorption, the flaxseeds were stored in sealed paper bags. Every sampling session involved collecting fifty intact and undamaged flaxseeds from each variety. Following acquiring hyperspectral images, they were immediately dispatched to the Gansu Academy of Agricultural Sciences in China to analyze protein, oil content, linoleic acid, and lignan for each variety.

**Table 1:** Flaxseed varieties.

No.	Variety	No.	Variety	No.	Variety
1	Onyc	11	Hua Ya 5	21	Yi Ya 3
2	Shuang You Ma 1	12	Hua Ya 6	22	Ba Ya 18
3	Shuang Ya 12	13	Ding Ya 17	23	Ba 14
4	Shuang Ya 14	14	Hei Ya 2	24	901 Ba Ya 15
5	Shuang Ya 15	15	Ning Ya 10	25	139 Ba Ya 17
6	Zhang Ya 3	16	Ba 9	26	Hua Ya 1
7	Ba 6	17	Ba 11	27	Hua Ya 2
8	Ba 5	18	Gan Ya 3	28	Hua Ya 3
9	Ba 4	19	Yan Za 10	29	Hua Ya 4
10	Ba 3	20	Jin Ya 7	30	Ba 2

### 2.2 Hyperspectral image capture

#### 2.2.1 Hyperspectral imaging system

**Figure 1:**
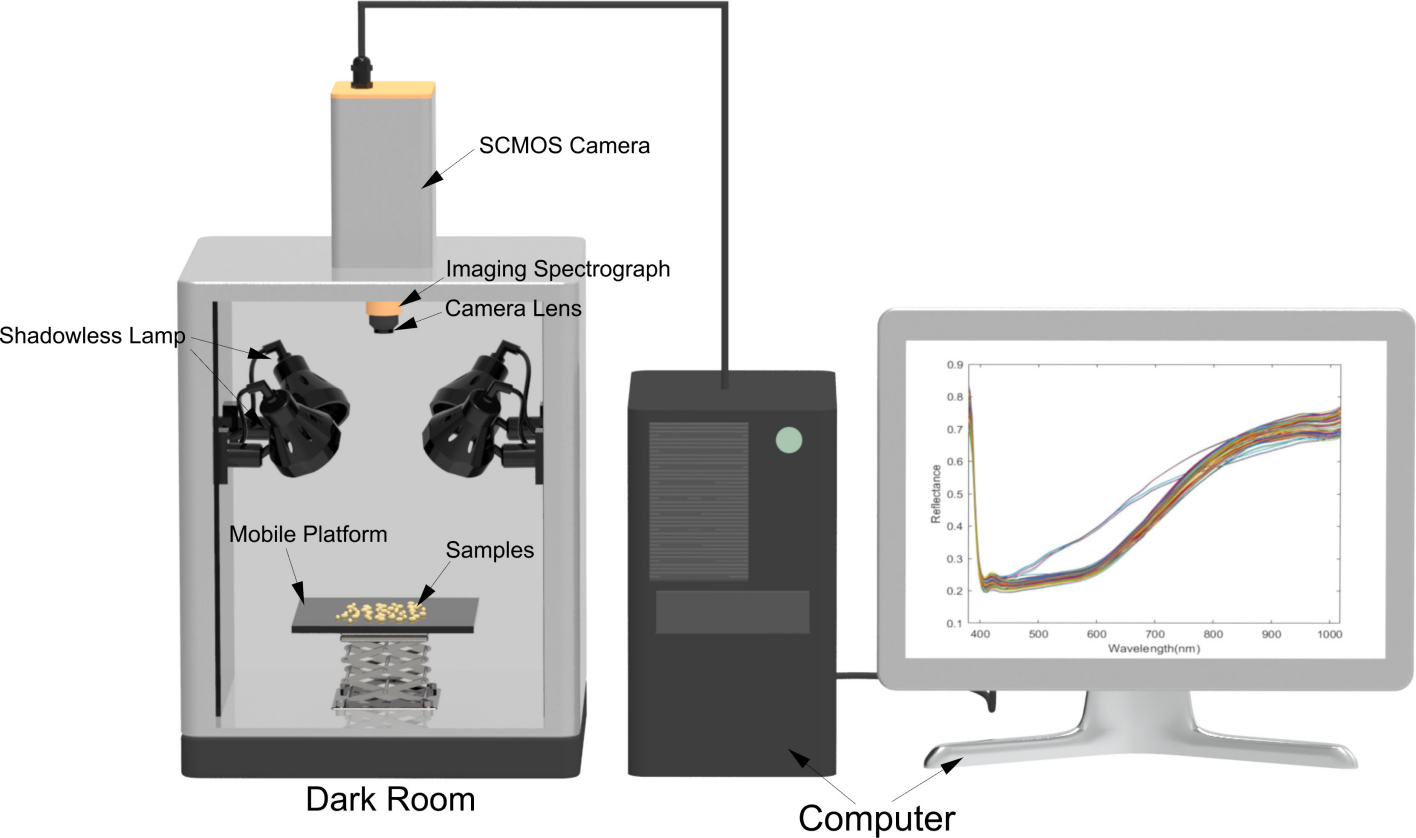
The hyperspectral imaging system.

The Gaia Field portable hyperspectral system (Sichuan Dualix Spectral Imaging Technology Co., Ltd) is shown in **Figure 1**, which includes GaiaField-V10E hyperspectral camera, 2048×2048 pixels imaging lens, HSI-CT-150×150 standard whiteboard (PTFE), HSIA-DB indoor imaging dark box, four groups of shadowless lamp light source, HSIA-TP-L-A tripod rocker set, and hyperspectral data acquisition software Spec View. The spectral range is 380-1018 nm, spectral bands are 320, spectral resolution is 2.8 nm, the numerical aperture is F/2.4, slit size is 30 μm× 14.2 μm, the detector is SCMOS, and the imaging mode is built-in push-scan, autofocus, and dynamic range is 14 bits. The core components of the hyperspectral equipment include a standardized light source, a spectral camera, an electronically controlled mobile platform, a computer, and control software. The working principle is that the system adopts the push-scan imaging mode, the surface array detector and the imaging spectrometer are combined, and under the drive of the scanning control electric moving platform, the slit of the imaging spectrometer and the focal plane of the imaging lens undergoes relative motion, the detector collects real-time information relative to the line target, and finally splices into a complete cube of data.

#### 2.2.2 Image acquisition and calibration

Enact the hyperspectral instrument switch and the dark box light source before image acquisition. Allow a 30-minute warm-up period, then configure the instrument parameters, setting the camera exposure time to 49ms, gain to 2, frame rate to 18.0018Hz, and forward speed to 0.00643cm/s. We have selected a total of 30 varieties of flaxseed; for each variety of hyperspectral images were collected a total of three times, each time from the corresponding varieties of randomly selected 50 seeds placed in the dark box on the mobile platform, as shown in **Figure 1**, and then these 50 seeds as the same ROI, to get an average spectral curve of these 50 seeds. After one acquisition for each variety, the sample under test was re-poured into the sample bag and shaken manually. Then, 50 seeds were randomly taken out for the subsequent image acquisition of that variety, repeated three times to get three average spectral curves and a total of 150 seeds were scanned. Ninety acquisitions were made for 30 varieties, with 4,500 seeds scanned, and 90 average spectral curves were obtained. After completing the acquisition, the original hyperspectral images underwent black-and-white correction to eliminate dark current noise introduced by the camera. (Wang et al., 2022). The black-and-white correction formula is shown in **Equation (1)**:


(1) $$I_{c}=\frac{I_{raw}-I_{dark}}{I_{white}-I_{dark}}$$

Where I_raw_ is the raw image, I_white_ is the white reference image, I_dark_ is the dark reference image, and I_c_ is the calibrated image.

**Figure 2:**
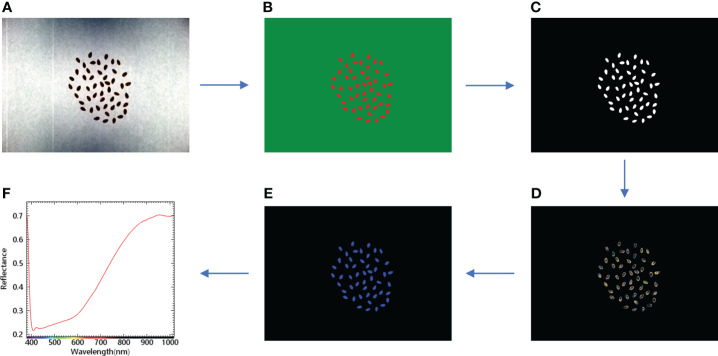
Sample hyperspectral image classification mask and spectral extraction flowchart. **(A)** Hyperspectral image; **(B)** Classification image; **(C)** Mask image; **(D)** Application mask image; **(E)** Region of interest image; **(F)** Average spectral curve.

In order to extract the spectral information from the corrected hyperspectral image, the 50 flax seed region in a single image was used as the region of interest, and the spectral information was extracted, as shown in **Figure 2**. Firstly, the regions of interest (ROIs) of flax seeds and background were created separately in ENVI5.3 software, and then according to the different ROIs, the flax seeds and background were classified using support vector machine (SVM) in supervised classification and transformed into vectors, followed by masking process and transformed into mask images. Applying the mask image to the original hyperspectral image separates the hyperspectral image of all the flaxseed sample regions from the background to get the region of interest for the whole sample. Finally, it calculates the average of the spectra of all the flaxseeds on the hyperspectral image as the spectrum of that sample.

### 2.3 Sample Content Determination and Segmentation

The protein, oil content, linoleic acid, and lignan contents of 30 varieties of flaxseed were determined by the Gansu Academy of Agricultural Sciences in China. Sample set partitioning based on joint X - Y distances (SPXY) (Liu et al., 2011) was employed to allocate flaxseed protein, oil content, linoleic acid, and lignan into modeling and prediction sets at a 2:1 ratio. The reasonableness of the sample division was assessed by calculating the samples’ maximum, minimum, average, and standard deviation in the training and prediction sets (Shao et al., 2020). The results are shown in **Table 2**. The maximum and minimum values of the training set for protein, oil content, and lignan included the prediction set, and the minimum values of the training set for linoleic acid and the prediction set were almost the same. Therefore, the overall division of the sample set is deemed reasonable.

**Table 2:** Flaxseed protein, oil content, linoleic acid, and lignan sample set contents.

Sample set	Protein		Oil content		Linoleic acid		Lignan	
	Cal	Pre	Cal	Pre	Cal	Pre	Cal	Pre
Number of samples	60	30	60	30	60	30	60	30
Maximum (%)	28.46	27.76	40.9	40.5	13.81	13.58	11.06	8.39
Minimum (%)	23.01	23.07	33.38	34.65	9.93	9.92	4.79	5.67
Average (%)	25.1	25.21	36.5	36.4	11.96	11.93	8.14	7.38
Standard deviation	1.54	1.28	1.62	1.5	0.86	0.82	1.49	0.71

### 2.4 Spectral preprocessing methods

During the acquisition of raw spectral data, it is often subject to various noise interferences, such as instrumental noise and environmental interference. In order to improve the quality and analyzability of the data, the extracted spectral information better reflects the changes in the sample curves to ensure that accurate and reliable results are obtained when building predictive models or conducting analyses. Therefore, it is necessary to pre-process the raw spectra to eliminate the noise as much as possible or reduce the influence of other environmental factors on the spectral information. The study employed various preprocessing techniques (Savitzky-Golay (SG) smoothing, normalization, baseline, standard normal variable correction (SNV), moving average (MA), multiple scattering correction (MSC), and first-order derivative (1st Der)) on the raw flaxseed spectra (Aulia et al., 2023). SG is mainly used to achieve the effect of smoothing curves and reducing noise by fitting local polynomials to the original spectra using a sliding window; Normalize can normalizes the spectral data to the same scale, which usually scales the value of each wavelength to a value between 0 and 1. It is mainly used to eliminate intensity differences due to differences in spectral measurement instruments, measurement conditions, and other factors; Baseline is based on the principle of removing baseline fluctuations in the spectrum due to instrumental drift, background changes, and other reasons, and can be used to improve the accuracy of the data; SNV is standardized by calculating the ratio of the spectral value at each wavelength to the mean and standard deviation of all spectral values at that wavelength; The aim is to reduce the intensity differences in the spectra and highlight the chemical information; MA focuses on averaging the spectral data over a sliding window to reduce high-frequency noise and smooth the spectral curves; MSC is based on the principle of correcting for multiple scattering by comparing the spectral data with a selected reference spectrum. This includes fitting each spectrum to the mean using least squares regression and calculating the preprocessed data by decomposing the slope and intercept of the regression. The aim is to reduce the effect of multiple scattering and emphasize the chemical information to improve the accuracy of quantitative analysis; 1st Der is to perform first-order derivative operations on the spectral data to highlight the rate of change of the spectral lines, enhance the peaks and valleys in the spectra, and highlight spectral line features. Subsequently, a PLSR prediction model for the protein content of flaxseed was established based on the raw and pretreatment spectra, and the optimal pretreatment method was determined by model evaluation.

### 2.5 Feature band extraction methods

Various sources frequently disrupt raw spectral data acquisition. Since the full spectrum contains 320 wavelength variables, not all wavelengths are useful for the analysis task. Extracting characteristic wavelengths reduces data dimensions, eliminates redundancy, and enhances modeling efficiency and performance. This study employs the successive projections algorithm (SPA) and the competitive adaptive reweighted sampling (CARS) algorithm for wavelength feature extraction. SPA algorithm is a forward looping feature variable selection method, which is a method of selecting feature wavelengths by calculating the correlation between each wavelength and the target variable, which is capable of filtering out the invalid information and greatly reducing the influence of covariance among the data. SPA has intuition and simplicity for the downscaling and feature selection of spectral data, which makes the model easier to interpret and understand (Li et al., 2023). CARS is an innovative variable selection algorithm proposed by Li (Li et al., 2009). At the same time, CARS is also a commonly used method for selecting the characteristic wavelengths, which firstly utilizes the PLS model to screen the wavelengths with large regression coefficients and then optimally selects the wavelengths with the smallest root-mean-square error through ten-fold cross-validation A subset of wavelengths is selected through ten-fold cross-validation, and the most critical variable for the prediction target is selected as the wavelength. The CARS algorithm is more flexible and adaptive than the traditional weighting methods, which helps to retain more useful information. In addition, CARS can more fully consider the correlation between wavelengths, thus better reflecting the characteristics of the data. In hyperspectral data, the CARS algorithm helps select representative characteristic wavelengths more comprehensively, considering that there may be complex relationships between wavelengths (Xu et al., 2022).

### 2.6 Modeling methods

Partial least squares regression (PLSR) is a multivariate statistical method (Wang et al., 2019). PLSR models the spectral data by minimizing the covariance between the spectral data and the target variable. It achieves data downscaling by introducing latent variables and then regressing these latent variables on the target variables.

Support vector regression (SVR) can fit data quickly (Xiang et al., 2022), and it deals with nonlinear relationships by mapping the data into a high-dimensional space and then constructing a linear regression model in that space.

Principal component regression (PCR) models spectral data by downscaling them into principal components to explain the variance of the spectral data and predict the target variable (Mahesh et al., 2015).

Multiple linear regression (MLR) is a conventional linear regression method that establishes the relationship between multiple independent variables and the dependent variable. In MLR, each wavelength is treated as a predictor variable, and the model tries to find a linear combination between these variables to fit the target variable best. However, MLR modeling only applies when the number of variables is less than the number of samples. Consequently, in this study, only wavelengths extracted by CARS and SPA algorithms were used for modeling (Rajkumar et al., 2012).

### 2.7 Software and model assessment

**Figure 3:**
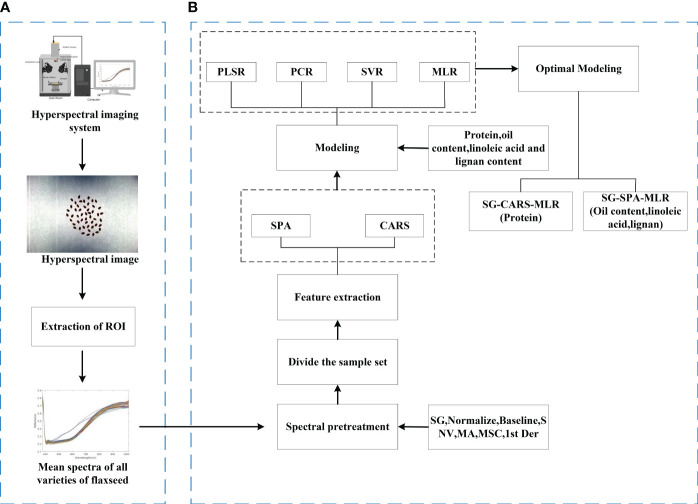
Experimental procedure. **(A)** Process of raw hyperspectral image acquisition and ROI extraction. **(B)** Spectral preprocessing, feature extraction, and modeling processes.


Besides using Spec view software for hyperspectral image acquisition and ENVI 5.3 for spectrum extraction, we utilized 3ds Max to construct a 3D model of the HSI system. Unscrambler X handled spectrum preprocessing and model building, while MATLAB R2021b extracted the featured wavelengths and plotted the waveforms. This paper assesses the model’s performance using various evaluation metrics, including the cross-validation correlation coefficient ( ${\text{R}}_{\text{cv}}^{2}$ ) and root mean square error (RMSECV), the calibration set correlation coefficient ( ${\text{R}}_{\text{c}}^{2}$ ) and root mean square error (RMSEC), and the prediction set correlation coefficient ( ${\text{R}}_{\text{p}}^{2}$ ) and root mean square error (RMSEP) (Zhang and Guo, 2020). The calculation process is detailed in Equation (2) and Equation (3). A well-performing model is characterized by high ${\text{R}}_{\text{cv}}^{2}$ , ${\text{R}}_{\text{c}}^{2}$ , or ${\text{R}}_{\text{p}}^{2}$ values and low RMSECV, RMSEC, or RMSEP values. These metrics gauge the model’s fitting and prediction capabilities, ensuring it excels in data fitting and new data prediction. The processing of the whole experiment is shown in **Figure 3**.

(2) $$R^{2}=1-\frac{\sum_{i-1}^{n}{\left(y_{i}-{\hat{y}}_{i}\right)}^{2}}{\sum_{i-1}^{n}{\left(y_{i}-{\overset{-}{y}}_{i}\right)}^{2}}$$

(3) $$RMSE=\sqrt{\frac{1}{n}\sum_{i-1}^{n}{\left(y_{i}-{\hat{y}}_{i}\right)}^{2}}$$

## 3 Results and analyses

### 3.1 Spectral characterization and selection of optimal preprocessing

**Figure 4:**
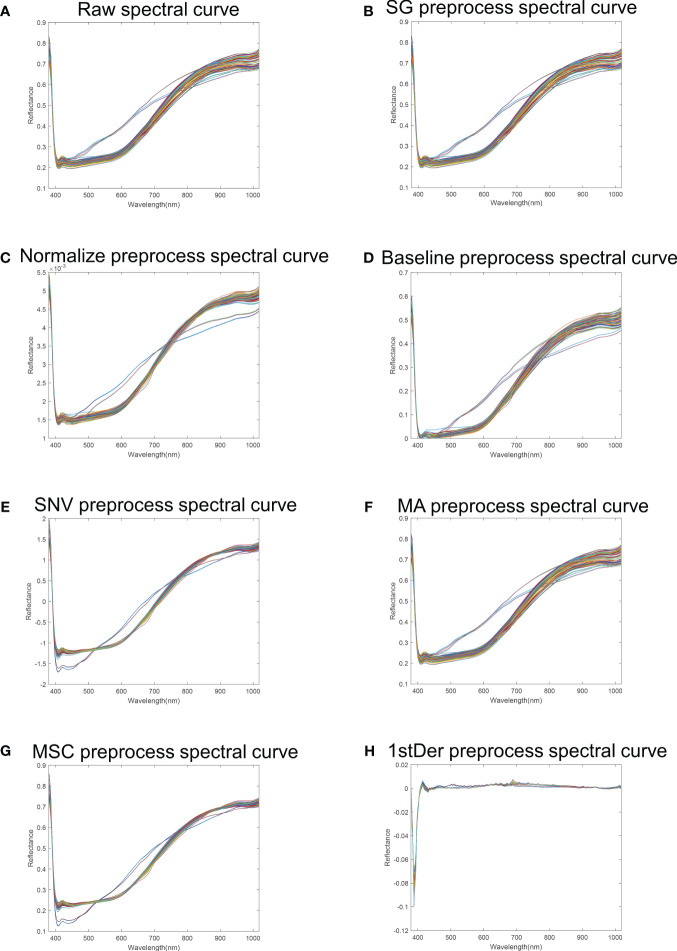
Flaxseed spectral reflectance curves. **(A)** Raw spectral curve of flaxseed; **(B)** SG preprocess spectral curve of flaxseed; **(C)** Normalize preprocess spectral curve of flaxseed; **(D)** Baseline preprocess spectral curve of flaxseed; **(E)** SNV preprocess spectral curve of flaxseed; **(F)** MA preprocess spectral curve of flaxseed; **(G)** MSC preprocess spectral curve; **(H)** 1stDer preprocess spectral curve.

**Figure 4** shows the average spectra of 30 different flaxseed varieties and the average spectra of 7 pre-treatments containing a total of 4,500 samples. As evident from **Figure 4A**, the average spectral profiles of various flaxseed varieties exhibit a consistent trend. However, notable deviations appear in the 450-800nm range, likely attributable to variations between flaxseed varieties. Further studies revealed that the average spectral profile of flaxseed has a significant reflectance peak at 420 nm, which is mainly caused by carotenoids (Yang et al., 2021). In addition, the spectral profile shows a clear upward trend in the range of 600-750 nm, which is attributed to the fact that this wavelength corresponds to the vibration of the N-H chemical bond of amino acids in the seeds (Xu et al., 2022). The absorption peak near 980 nm originates from the O-H stretching vibration, which is related to the structure of water molecules (Yu et al., 2014).

**Figure 5:**
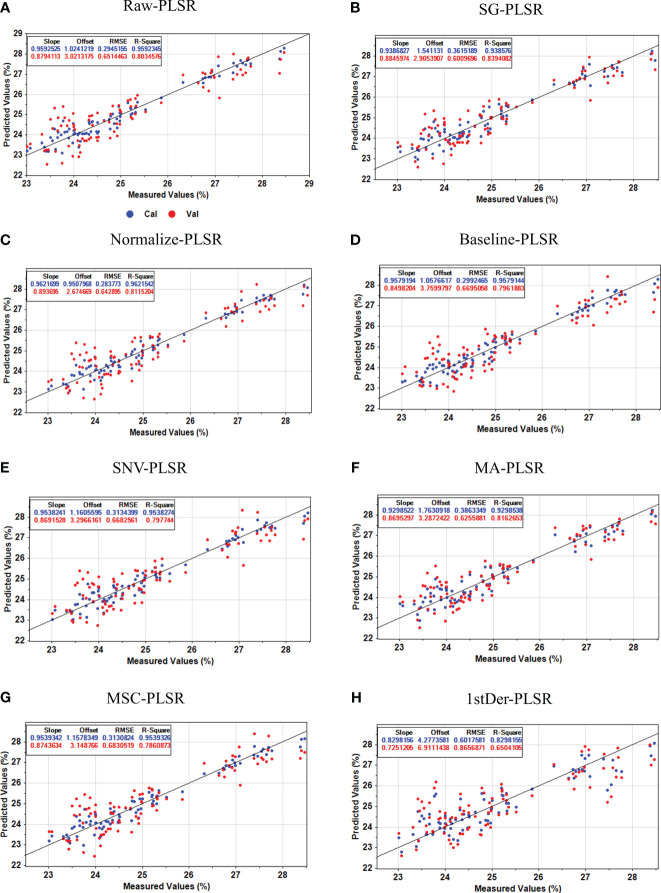
Protein content prediction results of the PLSR model based on different preprocesses. **(A)** Raw-PLSR; **(B)** SG-PLSR; **(C)** Normalize-PLSR; **(D)** Baseline-PLSR; **(E)** SNV-PLSR; **(F)** MA-PLSR; **(G)** MSC-PLSR; **(H)** 1stDer-PLSR.

To minimize the influence of noise and irrelevant information in spectral data, preprocessing of raw spectral information is essential. The Partial Least Squares Regression (PLSR) model comprehensively addresses the relationship between independent and dependent variables, even in scenarios of significant multicollinearity. The PLSR model for predicting flaxseed protein content identifies the best preprocessing method using stochastic cross-validation, employing Cross-validation set ${\text{R}}_{\text{cv}}^{2}$ and RMSECV as model evaluation metrics. **Figure 5** illustrates that, among the PLSR models predicting flaxseed protein content without pretreatment and with seven different pretreatment methods, the SG-PLSR model offered superior results, displaying a ${\text{R}}_{\text{cv}}^{2}$ value of 0.8394 and an RMSECV value of 0.6010. Thus, the SG pretreatment method was adopted for further feature extraction in predicting oil content, linoleic acid, and lignan content.

### 3.2 Results of feature extraction

**Figure 6:**
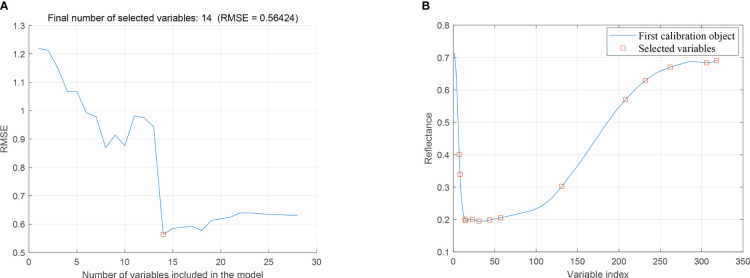
SPA extraction of feature variables. **(A)** Trend of RMSE with feature variables, **(B)** Distribution of preferred feature variables.

**Figures 6A, B** shows the wavelength distribution of flaxseed protein characteristics selected by the SPA algorithm, specifying the number of variables N = 1 to 30. When the variable is 14, the RMSE value is the smallest. Therefore, the final number of wavelengths selected is 14, accounting for 4.3% of the total number of wavelengths. These wavelengths, displayed in **Figure 6B**, correspond to the variables 391, 394, 405, 408, 424, 440, 465, 491, 640, 793, 842, 902, 990 nm and 1014 nm, respectively.

**Figure 7:**
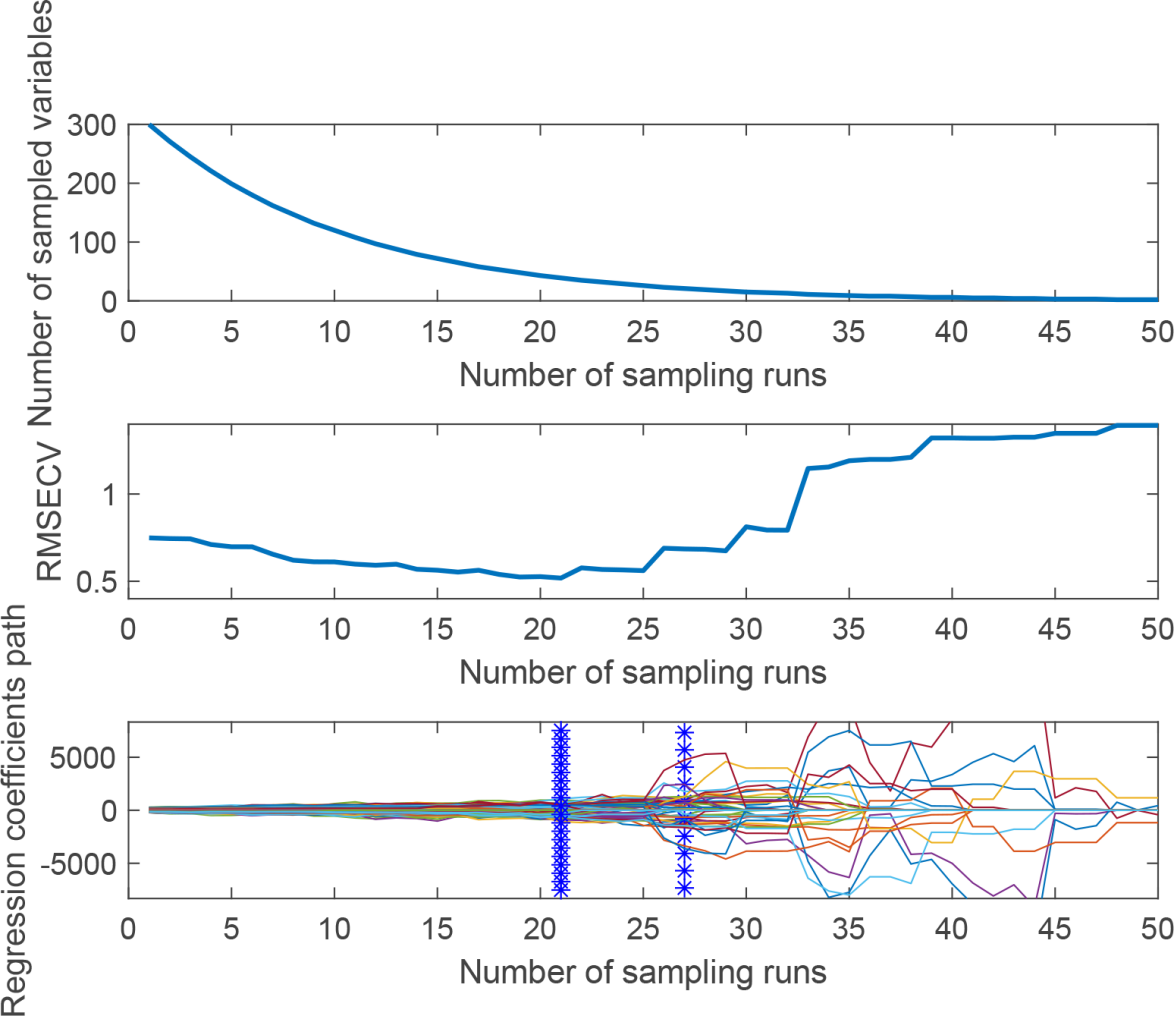
The process of extracting feature variables by CARS.

**Figure 7** shows the process of selecting the characteristic wavelengths of flaxseed proteins by the CARS algorithm, which includes the relationship between the number of sampling runs and the number of selected wavelength variables, the relationship between the RMSECV values and the relationship between the regression coefficients path. This figure illustrates that the efficiency of feature variable selection significantly improves from rough to fine screening with the increased number of sampling runs. Moreover, when the number of runs reached 21, RMSECV minimized, selecting 33 characteristic wavelengths crucial for predicting protein content. These wavelengths include 405, 408, 424, 438, 441, 465, 468, 494, 497, 501, 517, 519, 529, 569, 571, 574, 576, 593, 595, 598, 772, 844, 846, 880, 910, 931, 933, 958, 960, 986, 988, 1009 nm and 1014 nm, amounting to 10.3% of the total wavelength. This process indicates removing substantial irrelevant hyperspectral data and flaxseed protein content prediction in runs 1 to 20. The SPA and CARS methods were also used for characteristic wavelength extraction in subsequent oil content, linoleic acid, and lignan prediction modeling.

### 3.3 Results of modeling

#### 3.3.1 Modeling of hyperspectral prediction of protein content in flaxseed

**Figure 8:**
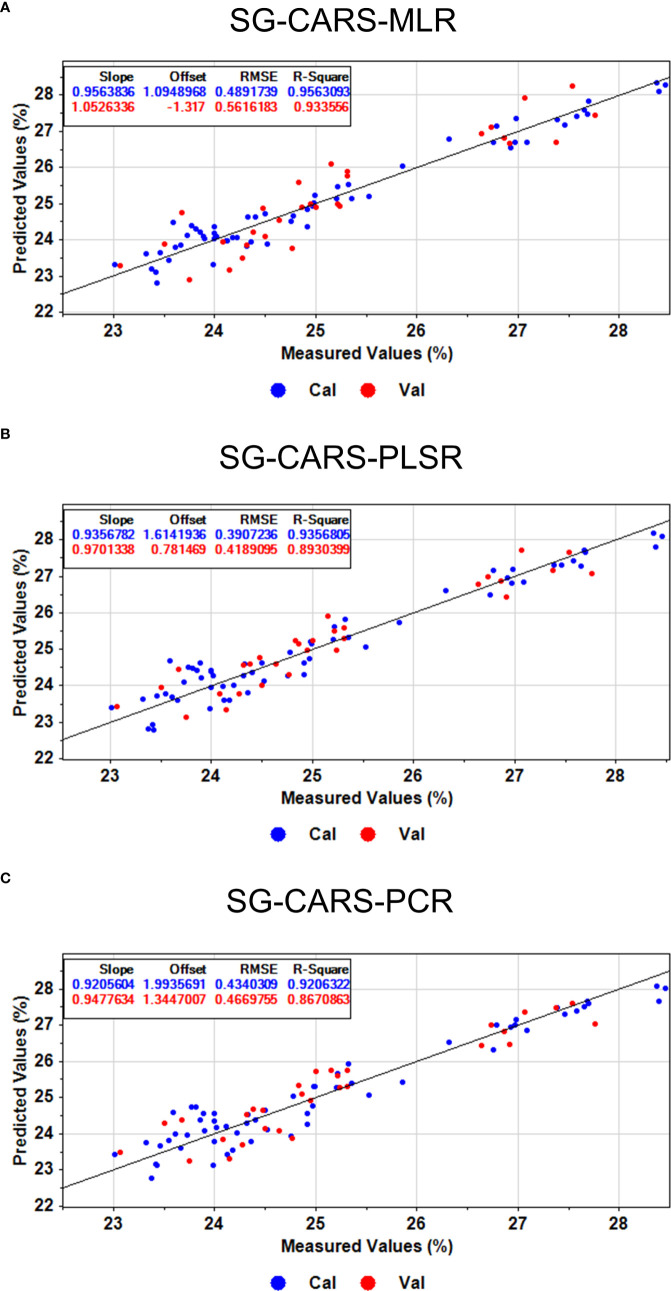
The optimal prediction of proteins based on **(A)** MLR, **(B)** PLSR, and **(C)** PCR models.

**Figure 9:**
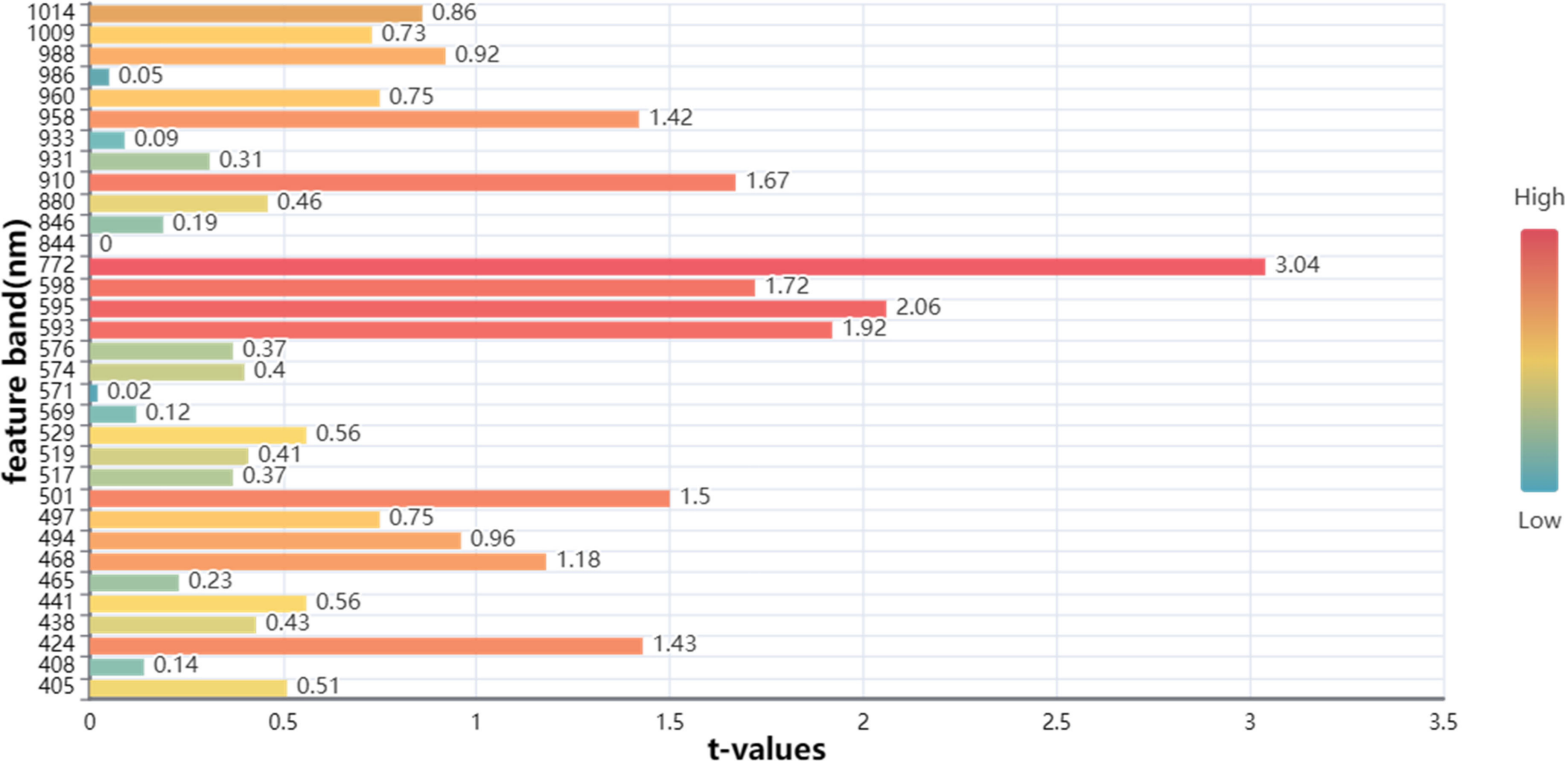
Significance map of MLR model for CARS extracted feature bands.

After determining the protein content of 30 flaxseed varieties, the original spectral data and the seven preprocessed data were combined with the actual protein content data to establish the PLSR prediction model of flaxseed protein. The cross-validation set ${\text{R}}_{\text{cv}}^{2}$ and RMSECV were used as evaluation indexes to determine the best preprocessing method. It was found that the model prediction of the data model after SG preprocessing was the best; therefore, the SG preprocessing method was used for the original spectral data to be preprocessed. Subsequently, we utilized both feature bands and full-band data extracted from the raw bands through SPA and CARS. These data were then input into regression models, including PLSR, SVR, PCR, and MLR, to predict flaxseed protein content. The results of these predictions are presented in **Table 3**. An analysis of the results in **Table 3** indicates that the PLSR, SVR, and PCR models, employing feature wavelengths extracted by the CARS algorithm, outperformed the models relying on full-band spectra. Specifically, they showed increased ${\text{R}}_{\text{p}}^{2}$ and decreased RMSEP values. Conversely, the SPA algorithm did not enhance the predictive performance and, in some cases, even reduced it. This observation suggests that SPA trims information redundancy but may also eliminate valuable information for accurate model predictions. In summary, different algorithms extracting distinct feature wavelengths significantly influence the effectiveness of the prediction models. The optimal model, SG-CARS-MLR, exhibited a training set ${\text{R}}_{\text{c}}^{2}$ of 0.9563, an RMSEC value of 0.4892%, a prediction set ${\text{R}}_{\text{p}}^{2}$ of 0.9336, and an RMSEP value of 0.5616%. The results for flaxseed protein content prediction in both the training and prediction sets are illustrated in **Figure 8A**. The other two models, SG-CARS-PLSR and SG-CARS-PCR (**Figures 8B, C**), also provided reasonably accurate protein content predictions, with ${\text{R}}_{\text{p}}^{2}$ values of 0.8930 and 0.8671, and RMSEP values of 0.4189% and 0.4670%, respectively. These findings confirm that the combination of HSI and the SG-CARS-MLR model delivers strong predictive performance for different flaxseed varieties’ protein content. Finally, characteristic bands associated with significant protein influence were identified using the SG-CARS-MLR model (**Figure 9**). Generally, when the absolute t-value surpasses a specific threshold (usually 2.0), it indicates the significant impact of a corresponding independent variable on the dependent variable. In this context, **Figure 8** shows that the bands at 595 and 772 nm exceed this threshold, signifying their substantial influence on the MLR model for protein content prediction.

**Table 3:** Protein prediction result table.

Modeling method	Feature extraction method	Number of feature variables	Cal		Pre	
			R^2^	RMSEC	R^2^	RMSEP
Protein						
PLSR	Non	320	0.9376	0.3848	0.7950	0.5800
	SPA	14	0.8933	0.5032	0.8197	0.5438
	CARS	33	0.9357	0.3907	0.8930	0.4189
SVR	Non	320	0.9546	0.3193	0.6366	0.9233
	SPA	14	0.9546	0.3193	0.6639	0.8845
	CARS	33	0.8632	0.6024	0.8061	0.7091
PCR	Non	320	0.6188	0.9512	0.4605	0.9408
	SPA	14	0.5479	1.0359	0.4282	0.9686
	CARS	33	0.9206	0.4340	0.8671	0.4670
MLR	Non	320	*	*	*	*
	SPA	14	0.9010	0.5597	0.9329	0.5642
	**CARS**	**33**	**0.9563**	**0.4892**	**0.9336**	**0.5616**

Represents that MLR modeling under 320 bands was not performed because MLR modeling is only applicable when the number of variables is less than the number of samples. Bold values indicate optimal model metrics.

#### 3.3.2 Hyperspectral prediction modeling of oil content, linoleic acid and lignan in flaxseed

**Figure 10:**
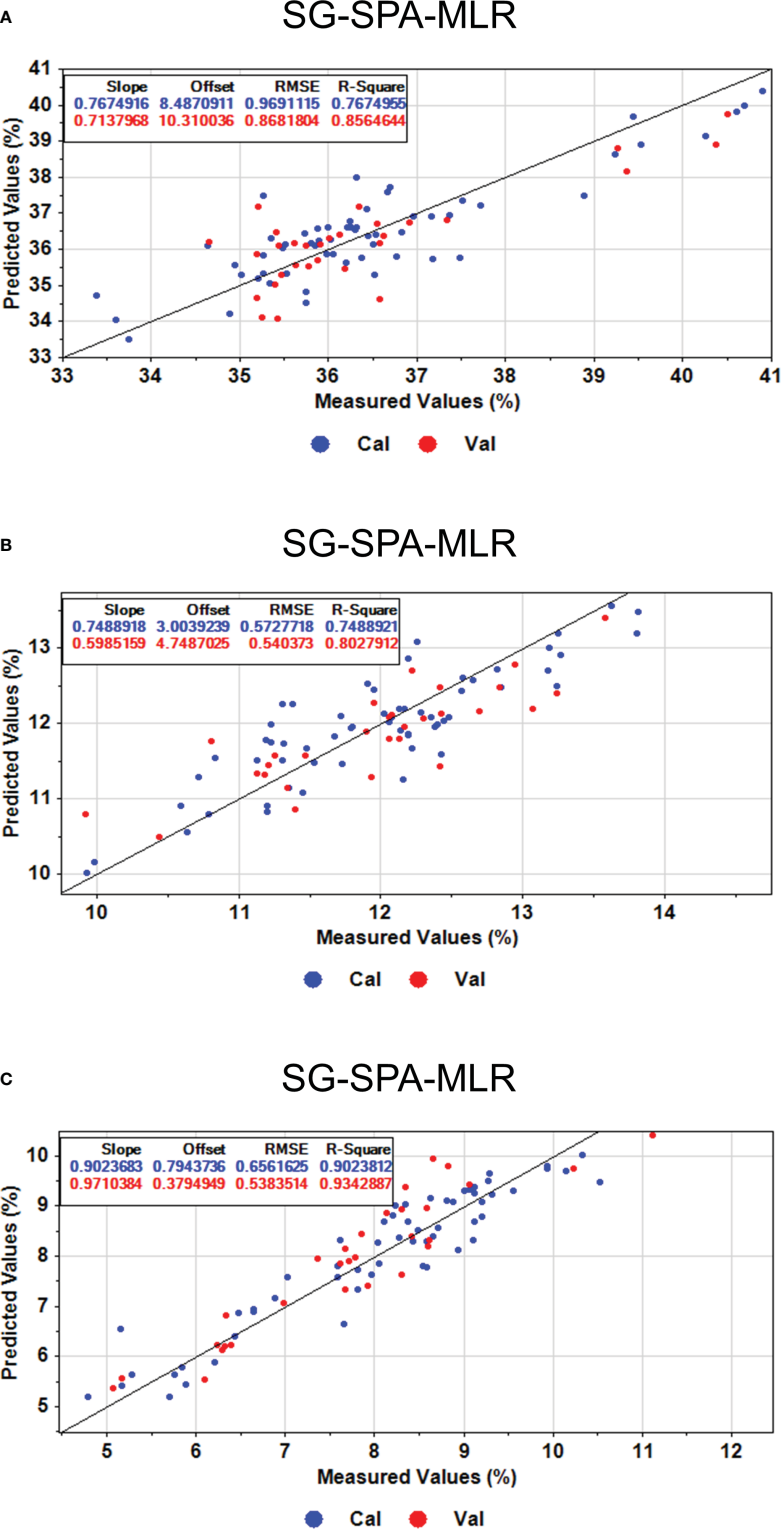
Predicted results of oil content, linoleic acid, and lignan content based on the optimal model SG-SPA-MLR. **(A)** Oil content prediction results. **(B)** Results of linoleic acid content prediction. **(C)** Prediction results of lignan content.

**Figure 11:**
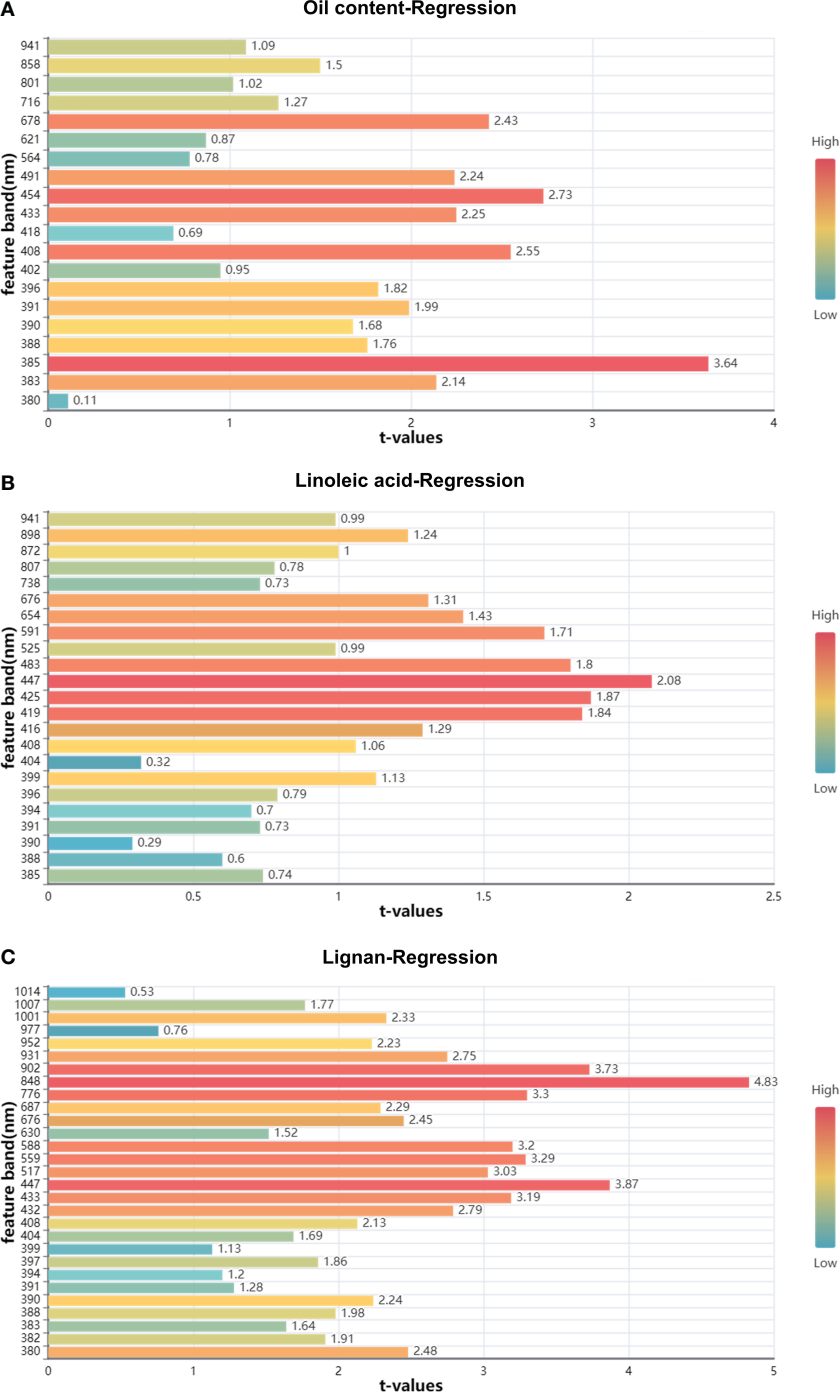
Significance map of MLR model for CARS extracted feature bands. **(A)** Significance map of the characteristic band of oil content; **(B)** Significance map of the characteristic band of linoleic acid; **(C)** Significance map of the characteristic band of lignan.

The prediction results for oil content, linoleic acid, and lignan content of flaxseed are presented in **Table 4**. The MLR model performs better than the PLSR, PCR, and SVR models. The ${\text{R}}_{\text{p}}^{2}$ values of PLSR, PCR, and SVR regression algorithms are all less than 0.8, indicating these models aren’t suitable for predicting the aforementioned contents in flaxseed. The extraction of feature wavelengths by SPA and CARS algorithms appears applicable to the MLR model. Specifically, the SG-SPA-MLR models perform better than SG-CARS-MLR in predicting oil content, linoleic acid, and lignan. For instance, the ${\text{R}}_{\text{p}}^{2}$ and RMSEP for oil content are 0.8565 and 0.8682%, and for linoleic acid are 0.8028 and 0.5404%, respectively. In contrast, the best model in literature predicting oil content for rapeseed seeds had an ${\text{R}}_{\text{p}}^{2}$ and RMSEP of 0.868 and 1.0698% (Li et al., 2023), respectively. Furthermore, lignan content was predicted with ${\text{R}}_{\text{p}}^{2}$ and RMSEP of 0.9343 and 0.5834%, respectively. Studies suggest that feature wavelengths derived from SPA and CARS algorithms enhance the predictive performance of MLR models, as observed in the prediction of moisture content of tobacco leaves (Sun et al., 2016) and the use of hyperspectral image technology for egg freshness detection (Wang et al., 2015). The scatter plots for the three types of flaxseed nutritional quality in both training and prediction sets are depicted in **Figure 10**, indicating the superior predictive performance of the SG-SPA-MLR model. Even though the ${\text{R}}_{\text{p}}^{2}$ for linoleic acid in the prediction set is 0.8028, the RMSEP is 0.5404%, affirming the model’s aptness for prediction. Finally, **Figure 11** highlights the importance of SPA-extracted feature bands in the MLR model. **Figures 11A, C** underscore the significance of these bands in predicting oil and lignin content. Notably, in **Figure 11C**, the MLR model predicts 18 feature bands with t-values greater than 2.0 in lignin content. These bands primarily appear around 470 nm (related to nitrogen content) (Li et al., 2022) and 800 nm (related to oxygen content) (Yuan et al., 2021), demonstrating the SG-SPA-MLR model’s superior prediction of lignan content.

**Table 4:** Oil content, Linoleic acid, and lignan prediction result table.

Modeling method	Feature extraction method	Number of feature variables	Cal		Pre	
			R^2^	RMSEC	R^2^	RMSEP
Oil content						
PLSR	Non	320	0.7401	0.826	0.6864	0.8397
	SPA	20	0.5218	1.1205	0.6058	0.9413
	CARS	10	0.6678	0.9339	0.6438	0.8948
SVR	Non	320	0.94	0.3952	0.5884	1.0305
	SPA	20	0.9399	0.3953	0.5884	1.0305
	CARS	10	0.94	0.3953	0.5884	1.0306
PCR	Non	320	0.5835	1.0458	0.6002	0.9481
	SPA	20	0.5917	1.0353	0.6077	0.939
	CARS	10	0.6866	0.9071	0.6572	0.8779
MLR	Non	320	*	*	*	*
	**SPA**	**20**	**0.7675**	**0.9691**	**0.8565**	**0.8682**
	CARS	10	0.6876	1.0022	0.8532	0.8779
Linoleic acid						
PLSR	Non	320	0.7204	0.4550	0.5502	0.5497
	SPA	20	0.6871	0.4813	0.5490	0.5504
	CARS	16	0.6404	0.5160	0.4495	0.6081
SVR	Non	320	0.9461	0.1977	0.7363	0.4516
	SPA	20	0.9462	0.1977	0.7362	0.4516
	CARS	16	0.9462	0.1977	0.7362	0.4516
PCR	Non	320	0.6474	0.5110	0.5381	0.557
	SPA	20	0.4604	0.6320	0.3418	0.6649
	CARS	16	0.6564	0.5043	0.4381	0.6143
MLR	Non	320	*	*	*	*
	**SPA**	**20**	**0.7489**	**0.5728**	**0.8028**	**0.5404**
	CARS	16	0.6740	0.5803	0.7286	0.6340
Lignan						
PLSR	Non	320	0.8597	0.5562	0.6626	0.8057
	SPA	29	0.5404	1.0067	0.5103	0.9707
	CARS	24	0.6362	0.8957	0.5475	0.9331
SVR	Non	320	0.9761	0.2688	0.6136	0.9082
	SPA	29	0.8464	0.6738	0.5177	1.0478
	CARS	24	0.9400	0.3953	0.5884	1.0306
PCR	Non	320	0.3959	1.1542	0.5105	0.9705
	SPA	29	0.5387	1.0086	0.4346	1.0430
	CARS	24	0.6249	0.9094	0.4880	0.9926
MLR	Non	320	*	*	*	*
	**SPA**	**29**	**0.9024**	**0.6562**	**0.9343**	**0.5384**
	CARS	24	0.7635	0.9455	0.8285	0.8697

Represents that MLR modeling under 320 bands was not performed because MLR modeling is only applicable when the number of variables is less than the number of samples. Bold values indicate optimal model metrics.

This project employs HSI technology within the 380-1018nm spectral range to gather data from flax seeds. The PLSR model cross-validation is then utilized to select the optimal pre-processing method, SG. Subsequently, characteristic wavelengths are extracted employing SPA and CARS algorithms. Finally, the spectral data corresponding to these characteristic wavelengths are combined with the protein, oil content, linoleic acid, and lignan acquired from the flax seeds through biochemical methods. This integration constructs four nutritional quality prediction models (SG-CARS/SPA-MLR) for rapid and non-destructive testing. The models achieve a prediction accuracy exceeding 0.93 for protein and lignan content, surpassing 0.85 for oil content. Although the linoleic acid content prediction accuracy is slightly lower, it still exceeds 0.80. These results fully address the requirements of practical production for rapid, non-destructive detecting of the nutritional quality of flaxseed grain.

## 4 Conclusions

The protein, oil content, linoleic acid, and lignan are crucial indicators for evaluating the quality of flaxseed. This study aimed to construct a model for the rapid and non-destructive detection of these components in flaxseed using HSI technology. Through experimental comparisons of various spectral image preprocessing methods and feature wavelength extraction algorithms, the preferred model achieved swift and non-destructive detection of protein, oil content, linoleic acid, and lignan in flaxseed grains, yielding better results. This research introduces a novel method for the future investigation of rapid, non-destructive, and high-precision detection of nutritional quality in different flaxseed varieties, enhancing the efficiency of screening and evaluating flax germplasm resources. The study’s results hold positive practical significance for the sustainable development of the flax industry and the selection and breeding of high-quality flax varieties.

## Data availability statement

The original contributions presented in the study are included in the article/supplementary material. Further inquiries can be directed to the corresponding author.

## Author contributions

DZ: Conceptualization, Data curation, Methodology, Software, Writing – original draft. JH: Conceptualization, Investigation, Resources, Supervision, Writing – review & editing. CL: Funding acquisition, Resources, Supervision, Writing – review & editing. JZ: Data curation, Resources, Writing – review & editing. YQ: Data curation, Resources, Writing – review & editing.

## References

[B1] Aulia R. Amanah H. Z. Lee H. Kim M. S. Baek I. Qin J.. (2023). Protein and lipid content estimation in soybeans using Raman hyperspectral imaging. Front. Plant Sci. 14. doi: 10.3389/fpls.2023.1167139 PMC10436576 37600204

[B2] Bjorgan A. Randeberg L. L. (2015). Towards real-time medical diagnostics using hyperspectral imaging technology. Eds. Brown J. Q. Deckert V. (Munich, Germany: European Conference on Biomedical Optics), 953712. doi: 10.1117/12.2184155

[B3] Dang Z. Zhao L. (2008). Application of the near infrared reflectance spectroscopy (NIRSin analyzing flaxseed germplasm quality. Acta Agric. Boreali-Occident. Sin. 17, 110–113.

[B4] Feng X. Li G. Song J. Shao H. (2016). Determination of lignan in flaxseed by HPLC. Anim. Husb. Feed Sci. 37, 17–18+22. doi: 10.16003/j.cnki.issn1672-5190.2016.12.005

[B5] Goyal A. Sharma V. Upadhyay N. Gill S. Sihag M. (2014). Flax and flaxseed oil: an ancient medicine & modern functional food. J. Food Sci. Technol. 51, 1633–1653. doi: 10.1007/s13197-013-1247-9 25190822 PMC4152533

[B6] Hu H. Wang T. Wei Y. Xu Z. Cao S. Fu L.. (2023). Non-destructive prediction of isoflavone and starch by hyperspectral imaging and deep learning in Puerariae Thomsonii Radix. Front. Plant Sci. 14. doi: 10.3389/fpls.2023.1271320 PMC10634472 37954990

[B7] Jiang M. Li Y. Song J. Wang Z. Zhang L. Song L.. (2023). Study on black spot disease detection and pathogenic process visualization on winter jujubes using hyperspectral imaging system. Foods 12, 435. doi: 10.3390/foods12030435 36765962 PMC9914266

[B8] Katare C. Saxena S. Agrawal S. Prasad G. Bisen P. S. (2012). Flax seed: a potential medicinal food. J. Nutr. Food Sci. 2, 120–127.

[B9] Li H. Liang Y. Xu Q. Cao D. (2009). Key wavelengths screening using competitive adaptive reweighted sampling method for multivariate calibration. Anal. Chim. Acta 648, 77–84. doi: 10.1016/j.aca.2009.06.046 19616692

[B10] Li X. Peng F. Wei Z. Han G. Liu J. (2023). Non-destructive detection of protein content in mulberry leaves by using hyperspectral imaging. Front. Plant Sci. 14. doi: 10.3389/fpls.2023.1275004 PMC10602742 37900759

[B11] Li M. Zhu X. Li W. Tang X. Yu X. Jiang Y. (2022). Retrieval of nitrogen content in apple canopy based on unmanned aerial vehicle hyperspectral images using a modified correlation coefficient method. Sustainability 14, 1992. doi: 10.3390/su14041992

[B12] Liu F. Jin Z. L. Naeem M. S. Tian T. Zhang F. He Y.. (2011). Applying near-infrared spectroscopy and chemometrics to determine total amino acids in herbicide-stressed oilseed rape leaves. Food Bioprocess Technol. 4, 1314–1321. doi: 10.1007/s11947-010-0445-y

[B13] Lu Y. Jia B. Yoon S.-C. Zhuang H. Ni X. Guo B.. (2022). Spatio-temporal patterns of Aspergillus flavus infection and aflatoxin B1 biosynthesis on maize kernels probed by SWIR hyperspectral imaging and synchrotron FTIR micro spectroscopy. Food Chem. 382, 132340. doi: 10.1016/j.foodchem.2022.132340 35139463

[B14] Ma C. Ren Z. Zhang Z. Du J. Jin C. Yin X. (2021). Development of simplified models for nondestructive testing of rice (with husk) protein content using hyperspectral imaging technology. Vib. Spectrosc. 114, 103230. doi: 10.1016/j.vibspec.2021.103230

[B15] Ma J. Sun D.-W. Pu H. Cheng J.-H. Wei Q. (2019). Advanced techniques for hyperspectral imaging in the food industry: principles and recent applications. Annu. Rev. Food Sci. Technol. 10, 197–220. doi: 10.1146/annurev-food-032818-121155 30633569

[B16] Mahesh S. Jayas D. S. Paliwal J. White N. D. G. (2015). Comparison of partial least squares regression (PLSR) and principal components regression (PCR) methods for protein and hardness predictions using the near-infrared (NIR) hyperspectral images of bulk samples of Canadian wheat. Food Bioprocess Technol. 8, 31–40. doi: 10.1007/s11947-014-1381-z

[B17] Meng D. Ji Z. Ren L. (2001). Determination of linoleic acid in grain by underivatized high performance liquid chromatography. Anal. Test. Technol. Instrum. 71–74.

[B18] Mueller K. Eisner P. Kirchhoff E. (2010). Simplified fractionation process for linseed meal by alkaline extraction – Functional properties of protein and fiber fractions. J. Food Eng. 99, 49–54. doi: 10.1016/j.jfoodeng.2010.01.036

[B19] Oomah B. D. (2001). Flaxseed as a functional food source. J. Sci. Food Agric. 81, 889–894. doi: 10.1002/jsfa.898

[B20] Ozaki Y. (2021). Infrared spectroscopy—Mid-infrared, near-infrared, and far-infrared/terahertz spectroscopy. Anal. Sci. 37, 1193–1212. doi: 10.2116/analsci.20R008 33612556

[B21] Rajkumar P. Wang N. EImasry G. Raghavan G. S. V. Gariepy Y. (2012). Studies on banana fruit quality and maturity stages using hyperspectral imaging. J. Food Eng. 108, 194–200. doi: 10.1016/j.jfoodeng.2011.05.002

[B22] Ribeiro L. F. Peralta-Zamora P. G. Maia B. H. L. N. S. Ramos L. P. Pereira-Netto A. B. (2013). Prediction of linolenic and linoleic fatty acids content in flax seeds and flax seeds flours through the use of infrared reflectance spectroscopy and multivariate calibration. Food Res. Int. 51, 848–854. doi: 10.1016/j.foodres.2013.01.061

[B23] Shao Y. Wang Y. Xuan G. Gao C. Wang K. Gao Z. (2020). Visual detection of SSC and firmness and maturity prediction for feicheng peach by using hyperspectral imaging. Trans. Chin. Soc Agric. Mach. 51, 344–350.

[B24] Sun J. Zhou X. Wu X. Zhang X. Li Q. (2016). Identification of moisture content in tobacco plant leaves using outlier sample eliminating algorithms and hyperspectral data. Biochem. Biophys. Res. Commun. 471, 226–232. doi: 10.1016/j.bbrc.2016.01.125 26809097

[B25] Tian X. Fan S. Huang W. Wang Z. Li J. (2020). Detection of early decay on citrus using hyperspectral transmittance imaging technology coupled with principal component analysis and improved watershed segmentation algorithms. Postharvest Biol. Technol. 161, 111071. doi: 10.1016/j.postharvbio.2019.111071

[B26] Tu K. Wen S. Cheng Y. Xu Y. Pan T. Hou H.. (2022). A model for genuineness detection in genetically and phenotypically similar maize variety seeds based on hyperspectral imaging and machine learning. Plant Methods 18, 81. doi: 10.1186/s13007-022-00918-7 35690826 PMC9188178

[B27] Wang Y. Guo W. Zhu X. Liu Q. (2019). Effect of homogenization on detection of milk protein content based on NIR diffuse reflectance spectroscopy. Int. J. Food Sci. Technol. 54, 387–395. doi: 10.1111/ijfs.13948

[B28] Wang Z. Huang W. Tian X. Long Y. Li L. Fan S. (2022). Rapid and non-destructive classification of new and aged maize seeds using hyperspectral image and chemometric methods. Front. Plant Sci. 13. doi: 10.3389/fpls.2022.849495 PMC9127793 35620676

[B29] Wang Q. Zhou K. Wang C. Ma M. (2015). Egg freshness detection based on hyperspectral image technology. Adv. J. Food Sci. Technol. 7, 652–657. doi: 10.19026/ajfst.7.1623

[B30] Xiang Y. Chen Q. Su Z. Zhang L. Chen Z. Zhou G.. (2022). Deep learning and hyperspectral images based tomato soluble solids content and firmness estimation. Front. Plant Sci. 13. doi: 10.3389/fpls.2022.860656 PMC9108868 35586212

[B31] Xu Y. Wu W. Chen Y. Zhang T. Tu K. Hao Y.. (2022). Hyperspectral imaging with machine learning for non-destructive classification of Astragalus membranaceus var. mongholicus, Astragalus membranaceus, and similar seeds. Front. Plant Sci. 13. doi: 10.3389/fpls.2022.1031849 PMC9745075 36523615

[B32] Yadav P. K. Burks T. Frederick Q. Qin J. Kim M. Ritenour M. A. (2022). Citrus disease detection using convolution neural network generated features and Softmax classifier on hyperspectral image data. Front. Plant Sci. 13. doi: 10.3389/fpls.2022.1043712 PMC9768035 36570926

[B33] Yang J. Sun L. Xing W. Feng G. Bai H. Wang J. (2021). Hyperspectral prediction of sugarbeet seed germination based on gauss kernel SVM. Spectrochim. Acta A. Mol. Biomol. Spectrosc. 253, 119585. doi: 10.1016/j.saa.2021.119585 33662700

[B34] Yao S. Liao M. Kang J. Wei Z. Liu N. REn H. (2022). Optimization of simultaneous extraction of oil, protein and gum from flaxseed by enzyme – assisted three phase partitioning. China Oils Fats 47, 11–17. doi: 10.19902/j.cnki.zgyz.1003-7969.210239

[B35] Ye J. Jia H. Guo D. Yan W. Xie L. (2021). Establishment and applicant of near-infrared reflectance spectroscopy models for predicting protein, linolenic acid and lignan contents of flaxseed. Chin. J. Oil Crop Sci. 43, 353–360. doi: 10.19802/j.issn.1007-9084.2019308

[B36] Yoosefzadeh-Najafabadi M. Earl H. J. Tulpan D. Sulik J. Eskandari M. (2021). Application of machine learning algorithms in plant breeding: predicting yield from hyperspectral reflectance in soybean. Front. Plant Sci. 11. doi: 10.3389/fpls.2020.624273 PMC7835636 33510761

[B37] Yu H. Liu H. Wang N. Yang Y. Shi A. Liu L.. (2016). Rapid and visual measurement of fat content in peanuts by using the hyperspectral imaging technique with chemometrics. Anal. Methods 8, 7482–7492. doi: 10.1039/C6AY02029A

[B38] Yu K.-Q. Zhao Y.-R. Liu Z.-Y. Li X.-L. Liu F. He Y. (2014). Application of visible and near-infrared hyperspectral imaging for detection of defective features in loquat. Food Bioprocess Technol. 7, 3077–3087. doi: 10.1007/s11947-014-1357-z

[B39] Yuan Z. Ye Y. Wei L. Yang X. Huang C. (2021). Study on the optimization of hyperspectral characteristic bands combined with monitoring and visualization of pepper leaf SPAD value. Sensors 22, 183. doi: 10.3390/s22010183 35009724 PMC8747622

[B40] Zhang Y. Guo W. (2020). Moisture content detection of maize seed based on visible/near-infrared and near-infrared hyperspectral imaging technology. Int. J. Food Sci. Technol. 55, 631–640. doi: 10.1111/ijfs.14317

[B41] Zhang H. Hou Q. Luo B. Tu K. Zhao C. Sun Q. (2022). Detection of seed purity of hybrid wheat using reflectance and transmittance hyperspectral imaging technology. Front. Plant Sci. 13. doi: 10.3389/fpls.2022.1015891 PMC9554440 36247557

[B42] Zhang Z.-S. Wang L.-J. Li D. Li S.-J. Özkan N. (2011). Characteristics of flaxseed oil from two different flax plants. Int. J. Food Prop. 14, 1286–1296. doi: 10.1080/10942911003650296

[B43] Zhu S. Chao M. Zhang J. Xu X. Song P. Zhang J.. (2019). Identification of soybean seed varieties based on hyperspectral imaging technology. Sensors 19, 5225. doi: 10.3390/s19235225 31795146 PMC6929038

[B44] Zou Z. Chen J. Wu W. Luo J. Long T. Wu Q.. (2023). Detection of peanut seed vigor based on hyperspectral imaging and chemometrics. Front. Plant Sci. 14. doi: 10.3389/fpls.2023.1127108 PMC10010490 36923124

